# Supplementary material for: Waves of Maturation and Senescence in Micro-structural MRI Markers of Human Cortical Myelination over the Lifespan
Source: Cereb Cortex. 2018 Dec 27;29(3):1369–81. doi: 10.1093/cercor/bhy330 (PMC6373687; doi:10.1093/cercor/bhy330)
Supplement: Supplementary Data [file bhy330_supplemental_material_clean.docx]

Supplemental Material for:

**Waves of Maturation and Senescence in Micro-Structural MRI Markers of Human Cortical Myelination over the Lifespan**

Håkon Grydeland, Petra E. Vértes, František Váša, Rafael Romero-Garcia, Kirstie Whitaker, Aaron F. Alexander-Bloch, Atle Bjørnerud, Ameera X. Patel, Donatas Sedervicius, Christian K. Tamnes, Lars T. Westlye, Simon R. White, Kristine B. Walhovd, Anders M. Fjell^*^, Edward T. Bullmore^*^

^*^ These authors contributed equally to this work.

Contents

[Supplementary Methods 2](#_Toc527145810)

[Participants 2](#_Toc527145811)

[MRI Data Acquisition 3](#_Toc527145812)

[Creation of T1w/T2w Maps 4](#_Toc527145813)

[Intracortical Depth 6](#_Toc527145814)

[Correction for T2w Scan Differences 7](#_Toc527145815)

[Estimations of Growth Curves 8](#_Toc527145816)

[Test of Unimodality 12](#_Toc527145817)

[Comparison with the Histological Cytoarchitectural Map of von Economo and Koskinas 13](#_Toc527145818)

[*Pre-processing functional MRI* 14](#_Toc527145819)

[Network Analyses 15](#_Toc527145820)

[Statistical Analyses 17](#_Toc527145821)

[Supplementary Tables 18](#_Toc527145822)

[Supplementary Table 1 18](#_Toc527145823)

[Supplementary Figures 19](#_Toc527145824)

[Fig. S1 19](#_Toc527145825)

[Fig. S2 20](#_Toc527145826)

[Fig. S3 21](#_Toc527145827)

[Fig. S4 22](#_Toc527145828)

[Fig. S5 23](#_Toc527145829)

[Fig. S6 24](#_Toc527145830)

[Fig. S7 25](#_Toc527145831)

[Fig. S8 26](#_Toc527145832)

[Fig. S9 27](#_Toc527145833)

[Fig. S10 28](#_Toc527145834)

[Fig. S11 29](#_Toc527145835)

[Fig. S12 30](#_Toc527145836)

[Fig. S13 31](#_Toc527145837)

[Fig. S14 32](#_Toc527145838)

[Supplementary References 33](#_Toc527145839)

# Supplementary Methods

**Participants**

The Regional Committee for Medical and Health Research Ethics of South Norway approved the study. We drew the sample mainly from the first wave of 2 on-going longitudinal projects by the Research Group for Lifespan Changes in Brain and Cognition at the University of Oslo, namely *Neurocognitive Development*, and *Cognition and Plasticity through the Lifespan*. Participants were recruited through newspaper ads, among students and employees at the University of Oslo, and from local schools. Further details regarding recruitment and enrolment can be found elsewhere (Westlye et al. 2009; Tamnes et al. 2010; Tamnes et al. 2013). Participants under 12 years of age gave oral informed consent, while written informed consent was obtained from all participants from 12 years of age and from a parent or guardian for participants below 18 years of age. Parents of children younger than 16 years, and all participants aged 16 years or older, were screened with standardized health interviews at enrolment to ascertain eligibility. We required participants to be right-handed, fluent Norwegian speakers, and have normal or corrected to normal vision and hearing. Self-reported neurological or psychiatric conditions known to affect normal cerebral functioning, including clinically significant stroke, traumatic brain injury, untreated hypertension, diabetes, use of psychoactive drugs within the last 2 years, or subjective concerns about cognitive status including memory function, were used as exclusion criteria. All participants above 20 years of age scored < 16 on the Beck Depression Inventory (Beck and Steer 1987) and participants above 40 years of age scored ≥ 26 on Mini Mental State Examination (Folstein et al. 1975). A neuroradiologist evaluated and deemed all scans free of significant injuries or conditions. Based on the existence of a T1-weighted and a T2-weighted scan for each individual, and after quality control excluding a total of 18 participants due to overfolding movement articfacts in T1w, T2w, or both, or missing/incomplete T2w scan, we included 484 participants: 263 females (54.3%), mean age (SD) = 38.3 (22.5) years, median age= 34.6 years, age range = 8.2-85.4 years. Of these, 73 new participants were included relative to our previous work (Grydeland et al. 2013): 37 females (50.7%), mean age = 15.4, SD = 3.3, median age= 15.4, min-max age = 9.1-21.6. All participants underwent assessment of general cognitive abilities by the Wechsler Abbreviated Scale of Intelligence (WASI) (Wechsler 1999). Estimated mean full-scale intelligence quotient for the entire sample was 112.9 (SD = 9.9, range = 82–145). Supplementary Figure 1 shows the gender and mean full-scale intelligence quotient for females and males across age deciles.

**MRI Data Acquisition**

All scans were acquired using a 12-channel head coil on a 1.5-T Siemens Avanto scanner (Siemens Medical Solutions, Erlangen, Germany) at Oslo University Hospital Rikshospitalet. For each participant, the T1w and the T2w scans was acquired in the same session. The T1w volumes were acquired using a 3D T1w magnetization-prepared rapid gradient echo (MPRAGE) sequence with the following parameters; repetition time (TR) = 2400 ms, echo time (TE) = 3.61 ms, inversion time (TI) = 1000 ms, 8° flip angle (FA), bandwidth = 180 Hz/pixel, field of view (FOV) = 240 mm, matrix = 192 × 192 × 160, 1.25 × 1.25 × 1.2 mm voxels. The T2w volumes were acquired using a 3D T2w sampling perfection with application optimized contrasts using different flip angle evolutions (SPACE) sequence with the following parameters: TR = 3390 ms, TE = 388 ms, variable FA, bandwidth = 650 Hz/pixel, FOV = 256 mm, 1 mm isotropic voxels. One hundred seventy-four participants (53.4% female) were scanned with a 204 × 256 × 176 matrix (mean age (SD) = 35.2 (19.5), range = 8.2-60.6), while 310 participants (54.8% female) were scanned with a 256 × 256 × 176 matrix (mean age (SD) = 40.0 (23.9), range = 8.5-85.4). Both T1w and T2w scans were acquired sagittally. The functional images consisted of 5 minutes of T2*-weighted single-shot gradient echo planar imaging scans with the following parameters: TR = 3000 ms, TE = 70 ms, 90° FA, 28 axial slices, FOV = 64 mm, 3.4375 × 3.4375 × 4 mm voxels. Participants were asked to lie with their eyes closed without falling asleep.

**Creation of T1w/T2w Maps**

T1w/T2w ratio maps for each participant were created by running the T1w and T2w images through the Human Connectome Project (HCP) processing pipeline (Glasser et al. 2013), using scripts kindly made available online by HCP (<https://github.com/Washington-University/Pipelines>). Via the HCP pipeline, the T1w volumes were processed using the Freesurfer 5.3 suite (<http://surfer.nmr.mgh.harvard.edu)>, including intensity normalization, automated tissue segmentation, generation of white and pial surfaces, surface topology correction, and surface-based cortical thickness and mean curvature maps (Dale et al. 1999; Fischl, Sereno and Dale 1999; Fischl and Dale 2000; Fischl et al. 2002; Fischl, Salat, et al. 2004; Fischl, van der Kouwe, et al. 2004; Segonne et al. 2004). In the preprocessing step, we did not perform the gradient distortion correction as scanning was performed on a conventional scanner (Siemens Avanto) compared with the custom scanner used in the HCP (Glasser *et al.* 2013) (we tested this additional step for one participant, the output looked very similar). Data was gathered without field map, so no readout distortion correction was performed. As noted in Glasser *et al.* (2013), this step removes a fairly subtle readout distortion, most pronounced in regions with high B0 inhomogeneity due to magnetic susceptibility differences (orbitofrontal cortex and inferior temporal cortex especially) (van der Kouwe et al. 2008). However, interpretation of effects, or lack thereof, in these areas should be made with caution.

The T2w image was registered to the T1w image by using Freesurfer’s *bbregister*, a within-subject, cross-modal registration using a boundary-based cost function constrained to be 6 degrees of freedom (rigid body) (Greve and Fischl 2009). As in our previous work, but here via the HCP pipeline, the resulting linear transform was applied by use of FSL’s *applywarp* tool using spline interpolation in order to minimize the white matter and cerebrospinal fluid (CSF) contamination of GM voxels (Glasser and Van Essen 2011). The T1w volume was divided on the aligned T2w volume, creating a T1w/T2w ratio volume.

To estimate regional T1w/T2w ratio across the brain, we used a multi-modal parcellation of the cortex into 180 symmetrical areas per hemisphere (Glasser et al. 2016). The parcellation was mapped into each participant’s native space to minimize geometric deformation of the data by inverting the warp from each participant to a common surface using a non-rigid high-dimensional spherical averaging method to align cortical folding patterns (Fischl, Sereno, Tootell, et al. 1999). Multimodal Surface Matching (Robinson et al. 2014) was not performed as i) participants between 40 and 60 did not having a resting-state functional MRI scan, and ii) as the T1w/T2w maps were being used as a measure of interest, we did not want to include these data in the registration process, and thereby potentially influence the registration based on differences in T1w/T2w values.

**Intracortical Depth**

From the resulting 360 cortical surface regions, the T1w/T2w ratio was extracted using the *wb_command -volume-to-surface-mapping* using cubic splines interpolation. We sampled T1w/T2w values vertex-wise from the WM/GM boundary, from 9 intracortical depths at 10% intervals, and at the grey matter (GM)/CSF boundary (pial), resulting in 11 surfaces. Each surface was obtained by using the *-surface-cortex-layer* command, which takes effects of folding into account, and thus follows the cortical layers more closely. **Fig. S8** shows higher T1w/T2w values at lower depths. As the surfaces near the WM/GM boundary and the GM/CSF boundary, respectively, suffer more from partial voluming effects (Polimeni et al. 2010), that is voxels being composed of different tissue types, we focused on the 30-70% depths (Polimeni *et al.* 2010; Huntenburg et al. 2017). From these middle depths, we chose the depth in which the age-relationships showed the lowest residual sums of squares (across regions), which was the 70% depth from the WM/GM boundary (**Fig. S9)**. Although we cannot precisely determine cortical layers, and at this resolution we obtain information from more than one layer, the 70% depth corresponds (on average) to layer 3 (Solari and Stoner 2011). Layer 3 has intracortical projections, that is, horizontal corticocortical projections traveling within the grey matter of the cerebral cortex based on work in the dorsolateral prefrontal cortex (Kritzer and Goldman-Rakic 1995). Although all pyramidal neurons have connections within the cerebral cortex, the prominent source of distant intracortical projections arise mainly from pyramidal neurons within layers 2 and 3, and a sub-set of neurons in layers 5 and 6 (Solari and Stoner 2011). Further, layer 2 and superficial layer 3 have been shown to contain intrinsic pyramidal neuron projections (in prefrontal areas 9 and 46) which make long distance lateral connections to patches of tissue in layers 1-3. Laterally travelling fibers are primarily in layers 2 and superficial 3, with oblique descending collaterals through layer 3. Pyramidal neurons of layer 3 also give rise to prominent horizontal projections with periodic terminations in layers 1-3 (see for instance figure 17 in (Levitt et al. 1993)).

In each of the 360 regions, outliers within each region for a single participant, and across participants, were identified as values exceeding the following formula using first (Q1), and third (Q3) quantiles, and the interquartile range (IQR): Q1-IQR*1.5, and Q3+IQR*1.5. These values were excluded.

**Correction for T2w Scan Differences**

To take into account the minor difference in acquisition parameters for the T2w scans (174 participants were scanned with a 204 × 256 × 176 matrix, while 310 participants were scanned with a 256 × 256 × 176 matrix), we estimated the effect of matrix in the developmental part of the sample where participants of overlapping ages were scanned with either one of the matrices (n_204_ = 67 (47.7% females), mean age (SD) = 13.4 (2.9), min-max = 8.2-18 versus n_256_ = 72 (50% females), mean age (SD) = 13.7 (2.6), min-max = 8.5-18). The effect was estimated running a robust regression using the Andrews weight function and the default tuning constant of 1.339 as implemented in Matlab, yielding coefficient estimates that are approximately 95% as statistically efficient as the ordinary least-squares estimates (provided the response has a normal distribution with no outliers). Age, sex, and an interaction term of age × matrix dimension were included as covariates of no interest. The estimated effect was then added to the 174 participants scanned with the 204 × 256 × 176 matrix.

**Estimations of Growth Curves**

To fit age trajectories without assuming a specific shape, for instance, linear, or quadratic, of the lifespan relationship *a priori*, we chose to use penalized cubic B-splines (Wood 2006; Fjell et al. 2010). Eight piecewise cubic B-spline basis functions were used, which was the lowest possible value in which all 360 models produced a fit. **Fig. S10** shows a penalized cubic B-spline growth curve fitted to simulated data, and the 8 underlying basis functions, multiplied by their respective coefficients. The fitted growth curve stem from a weighted sum of these basis functions (see also Alexander-Bloch et al. (2014)). The knots were placed at quantiles of the distribution of unique age values. The value for the smoothing parameter yielding appropriate degree of smoothness was found using restricted maximum likelihood (REML) (Wood 2011). REML was chosen as REML appear less prone to local minima than the generalized cross validation (Wood 2011), which resulted in a smoother derivative, and thus milestone estimates less affected by minor variations. Note that this choice effectively imposes prior beliefs about the correct model. For instance, in the extreme case, a model passing through all data points would *a priori* be considered as not biologically plausible as it would not transition smoothly across the age range. Thus, although the model structure allows considerable flexibility, we chose to penalize excessively fluctuating models, i.e. that are not biologically plausible (Wood 2006). The median effective degrees of freedom was 5.7 (range = 3.5-6.9).

The derivative of the fitted penalized cubic B-spline growth curve was obtained by calculating the slope at each point along the curve using the *diff* function in R. Specifically, the derivative was taken as the differences ($\Delta y$) between the predicted growth curve (using the *predict* function) from i) one set of age values, and ii) a set of slightly increased (0.08 years) age values, divided by this 0.08 increment in age $(\Delta x)$: $\frac{\Delta y}{\Delta x}\mathrm{or}\frac{dy}{dx}$ . This calculation yielded a point estimate at each point along the growth curve. We wanted to know at which ages the increases or decreases were sufficient to be considered statistically significant. Therefore, we estimated 99.99% confidence interval of the derivative. This level was used in order to yield conservative estimates, as the confidence interval is correct at each point, but liberal if looking at several points (that is, not corrected for familywise error). These confidence intervals were obtained as implemented in *predict.gam* (Marra and Wood 2012) in R (https://cran.r-project.org), using the Bayesian posterior covariance matrix, and multiplying the derivatives (in matrix form, for each smooth term) with this covariance matrix, before multiplying this product with the derivatives. To obtain the standard error for the entire spline, and not for each of the basis functions that comprise the spline, the resulting values were summed across spline terms, and their square root taken.

Based on the confidence interval of the derivative, we extracted 3 curve features, or milestones, namely age at peak growth, age at onset of stability, and age at onset of decline. Peak growth age was defined as the maximum positive value. Onset of stability age was the first point showing a non-significant slope, that is, the age at which the lower confidence interval crossed zero for the first time. Onset of decline age was the point where the curve again showed a significant slope, that is, the age at which the upper confidence interval crossed zero. We also estimated cross-sectional measures of the rate of growth, and the rate of decline (**Fig. S1**). For the rate of growth, we extracted the derivative at peak growth age. The rate of decline was also extracted from the derivative, at age A in the following manner: the sum of i) onset decline age, for instance 60 years, and ii) the age at the halfway point (50%) between a) onset decline age, and b) maximum age in the sample, for instance: (85-60) × 0.5 = 12.5, which in this example would yield A = 60 + 12.5 = 72.5 years as the age of measurement. As the derivative is the slope between 2 points, all values were taken as the average between the value and the successive value. The procedure of estimating the spline, calculating the derivative and confidence intervals, and obtaining milestones was repeated for each of 360 regions.

The derivative CIs are important for the estimations of the milestone. Thus, to assess the trustworthiness of our parametric estimates, we also calculated the CI by using the boostrap method. Specifically, we drew 5000 random samples with replacements across participants, and calculated the growth curve and its derivative as above for each region. The 99% CIs were taken as the 0.05 and 0.995 percentile, respectively, of the resulting distribution of samples, at each point along the curve and the derivative, respectively.

To probe potential confounding effects of motion on the growth curves, we performed 2 post-hoc analyses. First, as a proxy measure of motion, we calculated temporal signal-to-noise ratio (tSNR) (Roalf et al. 2016) (QAscripts, v2) from same-session diffusion-weighted scans. These scans were available for all but two participants. We created a sub-sample by removing participants with tSNR below the 10^th^ percentile (**Supplemental Material, Fig. S13**, 49 participants, forming two distributions centred around 17 and 71 years, respectively). Second, we used a recently proposed (Rosen et al. 2018) index of image quality from the T1w scans themselves, namely the Euler number calculated during the FreeSurfer stream (Dale et al. 1999). Rosen et al. showed that the Euler number identified images scored “unusable” by human raters with a high degree of accuracy (area under the curve of 0.98 and 0.99 in testing sample 1 (n=533, mean (SD) age 15.1 (3.7) years), and testing sample 2 (n=242, mean (SD) age 41.4 (17) years)), respectively. FreeSurfer calculates one Euler number per hemisphere, and we used an average value across hemispheres per participant. Based on this average Euler number, we created another sub-sample by again removing participants below the 10^th^ percentile (**Supplementary Material, Fig. S13**), 49 participants, forming two distributions centred around 11 and 77 years, respectively). There was a 27% overlap with the excluded participants based on the tSNR. Based on these sub-samples, we re-estimated the milestones, and re-plotted the resulting curves across regions (as in **Fig. 1B**), and histograms (as in **Fig. 2B**) to assess how our main findings might have been influenced by the inclusion of motion-compromised scans (**Supplementary Material, Fig. S14**)).

**Test of Unimodality**

For each of the milestone distributions, we tested forunimodality using Hartigans' dip test statistic for unimodality (Hartigan and Hartigan 1985) in R using the diptest package. As the dip test yields very conservative p-values (Xu et al. 2014), we simulated P values from the unimodal distribution closest to the data (Tantrum et al. 2003) using the fpc package. An indication of non-unimodality, that is, at least bimodal distributions was deemed present if the tests were significant (P < 0.05). To further validate this result, we used an expectation–maximization (EM) algorithm to fit Gaussian finite mixture models with 1 and 2 components (a maximum of 2 components would provide proof of concept, and not be liable to over-fitting) as implemented in the R package mclust, and tested for best fit using a bootstrap likelihood ratio test with 10000 bootstraps. We fitted an EM algorithm (**Fig. 2B**), as employed in the R package mixtools which yielded parameters for mu and sigma, for visualization. The fit was qualitatively deemed good (except for the second mode in M3, which, however, clearly stood out), and we proceeded with exploratory analyses dichotomizing the 3 milestones. The age used to separate the two waves in each distribution was obtained by visual inspection of the point where the density functions crossed (the second mode in M3 was identified as the late wave).

To describe the cognitive functions of regions showing the different wave properties we used NeuroSynth (<http://neurosynth.org>), a data-driven tool that mines the published neuroimaging literature and provides terms most likely to be used in publications alongside specific MRI coordinates (Yarkoni et al. 2011). For this study we passed the two wave maps (early and late) for each milestone to the NeuroSynth decoder and visualised the top 25 terms related to the highlighted regions as a word cloud. The size and colour saturation of the words in **Fig. 2D** correspond to the frequencies associated with each term.

**Comparison with the Histological Cytoarchitectural Map of von Economo and Koskinas**

Following van den Heuvel et al. (2015) and Vertes et al. (2016), we explored the relationship between cortical histology and the three key milestones. To this end, based on Scholtens et al. (2016) and Solari and Stoner (2011), we assigned each of the 360 regions to 1 of 5 cytoarchitectonic types classified according to the scheme of von Economo and Koskinas (Triarhou 2007), reflecting the 5 structural types of isocortex, namely homotypic, or type 2, 3 and 4, and heterotypic, or type 1 agranular and type 5 granular. Again following Vertes et al. (2016), as the original classification of structural types does not discriminate between true six-layered isocortex, and mesocortex or allocortex, we defined two additional subtypes: limbic cortex which included the entorhinal, retrosplenial, presubicular and cingulate cortices, and thus primarily constitutes allocortex, and the insular cortex which contains granular, agranular and dysgranular regions, and is therefore not readily assigned a single structural type. Structural classes were manually assigned to each region in Scholtens et al.’s parcellation, based on Figure 3 in Solari and Stoner (2011), Figure 1F in Vertes et al. (2016), and anatomical landmarks. Each of the 360 used here was then assigned a class based on overlap with the Scholtens et al. parcellation. In cases of overlap with more than one class, the region was assigned the class with which it overlapped the most.

## **Pre-processing functional MRI**

A subset of the included participants also underwent functional MRI while resting. To compare and extend our structural network analyses on a subset of the participants between 34 and 72 years (age of stability), we chose participants similar in age, but lowering the threshold to 20 years of age to boost sub-sample size, as only 17 participants between 34 and 60 years underwent fMRI. A total of 13 fMRI scans were deemed to not meet the standards set for quality due insufficient whole brain coverage (10 in total), or movement (3 in total, 2 in the young adults between 20 and 40 years of age, and 1 in the elderly adults, absolute root-mean square above 1.5 mm was deemed excessive). These participants were excluded from the sub-sample, yielding a final functional connectivity sub-sample of 128 participants, centered around two means: mean (SD) age 28.6 (6) years (min-max age 20-40 years, n=73, 59% females), and mean (SD) age 64.5 (2.5) years (min-max age 60-72 years, n=55, 58% females).

Each 4D fMRI volume was skullstripped, and rigid-body aligned to the middle volume with spline interpolation via the mcflirt tool (Jenkinson et al. 2002) to correct for motion between volume acquisitions, saving the amount of rotation and translation in 3 dimensions, respectively, and then slice timing corrected, smoothed (8 mm full width at half maximum), and intensity normalized (a within-run whole-brain median intensity scaling to 10000), as implemented in FSL’s FMRI Expert Analysis Tool (Smith et al. 2004; Woolrich et al. 2009). After registering the functional volumes to the T1w volume using epi_reg and bbregister, the resulting transformations were used to bring the left and right lateral ventricle masks as segmented by FreeSurfer to each participant’s functional space, where the masks were eroded (thresholded at 0.5), and used to derive the average cerebrospinal fluid (CSF) signal per time point. The fMRI data were then despiked for motion artifacts using the BrainWavelet Toolbox v1.1 (Patel et al. 2014). The despiked time series were subsequently, together with the average CSF signal, and the 6 motion estimates and their first order temporal derivatives, entered in a linear regression, and the residuals saved for further analyses.

**Network Analyses**

**Structural network.** To investigate how the milestones, which are local in nature, might relate to global network properties of the brain, we constructed an anatomical network employing a covariance approach (Alexander-Bloch et al. 2013; Evans 2013). This approach allowed us to use the same data in the network analysis as we used for calculating the milestones. It has been shown that regions with high structural covariance or correlation are often involved in the same cognitive function and connected via WM pathways (Lerch et al. 2006). Specifically, we correlated T1w/T2w for each region with all other regions, across participants between the ages of maturity and decline onset in the global curve (see **Fig. 1B**; 34 and 72 years, that is, a period with relative stable T1w/T2w levels. This procedure yielded a 360×360 connectivity matrix, which was binarized employing a minimum spanning tree approach followed by global thresholding, retaining 10% of the strongest connections or edges (**Fig. S6A**) (Alexander-Bloch et al. 2010). From this model, we assessed degree and modularity, two of the most common and, for degree, interpretable network metrics. These analyses were carried out in Matlab (https://www.mathworks.com) using the Brain Connectivity Toolbox (Rubinov and Sporns 2010). Degree refers to the number of edges for each region (node). The degree distribution was fat-tailed (**Fig. S6C**), indicating the presence of high-degree regions, so-called hubs. Modularity measures community structure, or to what extent nodes aggregate into smaller groups hallmarked by being more interconnected between members of the groups than across groups. Here, modularity was calculated using the Louvain algorithm (Blondel et al. 2008), using consensus clustering (Sporns and Betzel 2016). The choice of resolution parameter for modular decomposition, γ, which usually is set to 1, was empirically defined by finding a local minimum for nodal versatility of modular affiliation (Shinn et al. 2017), to identify values of γ at which nodes are consistently assigned to the same module. After calculating versatility from γ = 0.1 to 4, with 0.01 increments, we chose the gamma which minimized versatility, here γ = 1.12. Based on the final community partition (**Fig. S6B)**, we also evaluated the intra- and intermodule degree, that is connections within a node’s community, and across other communities, respectively***.*** A network representation of the structural connectivity (**Fig. 4ii)** was visualised using NetworkX, version 2.1 (<https://networkx.github.io>).

Functional network. To further assess the relationship between the milestones, and brain network topology, we also created a functional network based on functional MRI scans at rest for a subset of the participants (aged 20 and 72 years, please see above for details). From the resulting pre-processed fMRI volumes (please see above for details), regional average time series were extracted for the 360 regions. The time series were then fed to a wavelet decomposition (Bullmore et al. 2004) to 4 wavelet scales using the maximal overlap discrete wavelet transform with a Daubechies 4 wavelet, performed by use of the brainwaver package (Achard et al. 2012) in R ([www.r-project.org/](http://www.r-project.org/)). We chose wavelet scale 1 frequency band (0.08-0.17 Hz) for further investigation, but also performed the analyses at the scale 2 frequency band (0.04 -0.08 Hz) to assess consistency. The wavelet correlation between every pair of regions yielded one 360 × 360 matrix of regional functional connectivity per participant. We then averaged these matrices across participants resulting in one 360 × 360 matrix of regional functional connectivity across the sub-sample. Similar network construction and analyses were performed as for the structural network, with focus on the total degree analysis. Thus, we constructed a binary graph by retaining the 10% strongest connections for this average functional connectivity network, using the minimum spanning tree approach. This network representation was fed to network analyses of community structure (again we chose the gamma which minimized versatility, here γ = 0.94), and total degree centrality.

**Statistical Analyses**

Relationships between various node measures (for instance average T1w/T2w, and milestones) were tested using Spearman’s rank correlation, except between rate of maturation and decline, where a linear regression was performed. To test for differences in rates of growth and decline, respectively, between early and late waves across the 3 milestones, we calculated 95% confidence intervals. In the cases where the confidence interval overlapped, we tested for differences between waves by Wilcoxon rank sum test. The same approach was used to test for differences in the degree measures between waves. To test for the differences across cytoarchitectonic classes, we employed Kruskal-Wallis tests by rank, a non-parametric alternative to an analysis of variance (ANOVA). Here, to account for the multiple tests performed, false discovery rate (FDR) correction was applied using the method of Benjamini, and Yekutieli (Benjamini and Yekutieli 2001) via the p.adjust function in R.

# Supplementary Tables

## Supplementary Table 1

| A |  | Rate (x 10^-2^) | | B | Degree | | | |
| --- | --- | --- | --- | --- | --- | --- | --- | --- |
|  |  | Maturation | Decline | Total | Intramodular | Intermodular | Total fMRI 1 | Total fMRI 2 |
| M1 | W1 | 1.56 [1.44,1.69] | -1.04 [-1.21,-0.90] | 11 [8,17] | 6 [5,9] | 5 [3,9] | 63 [51,76] | 59 [49,73] |
|  | W2 | 1.14 [1.09,1.19] | -0.76 [-0.84,-0.68] | 41 [37,45] | 21 [19,23] | 19 [17,22] | 31 [27,36] | 31 [27,36] |
| M2 | W1 | 1.31 [1.26,1-36] | -0.93 [-1.01,-0.85] | 35 [31,40] | 19 [17,21] | 16 [14,19] | 39 [34,44] | 39 [34,45] |
|  | W2 | 0.86 [0.79,0.93] | -0.40 [-0.48,-0.32] | 39 [31,47] | 20 [16,24] | 19 [14,24] | 24 [18,31] | 22 [17,29] |
| M3 | W1 | 1.45 [1.39,1.52] | -1.25 [-1.34,-1.16] | 31 [26,36] | 17 [15,20] | 14 [11,18] | 50 [43,57] | 47 [41,55] |
|  | W2 | 0.99 [0.94,1.04] | -0.40 [-0.47,-0.34] | 41 [35,46]^w^ | 21 [18,24]^x^ | 20 [17,23]^y^ | 24 [20,29] | 26 [21,31] |

A. Maturation and decline rate (cross-sectionally estimated) for each milestone (M1-M3) and wave (W1-W2). Confidence intervals were calculated using the adjusted bootstrap percentile method (10000 bootstraps replicates) using the R package *rcompanion*.

B. Mean and 95% confidence intervals for each milestone and wave.

^w^ = Wilcoxon rank sum test between wave 1 and 2: W = 13427, P = 0.006 (two-sided).

^x^ = W = 14224, P = 0.050.

^y^ = W = 13496, P = 0.006.

Total fMRI 1/2: Total degree derived from functional MRI scans at rest, for wavelet scale 1 and 2, respectively.

# Supplementary Figures


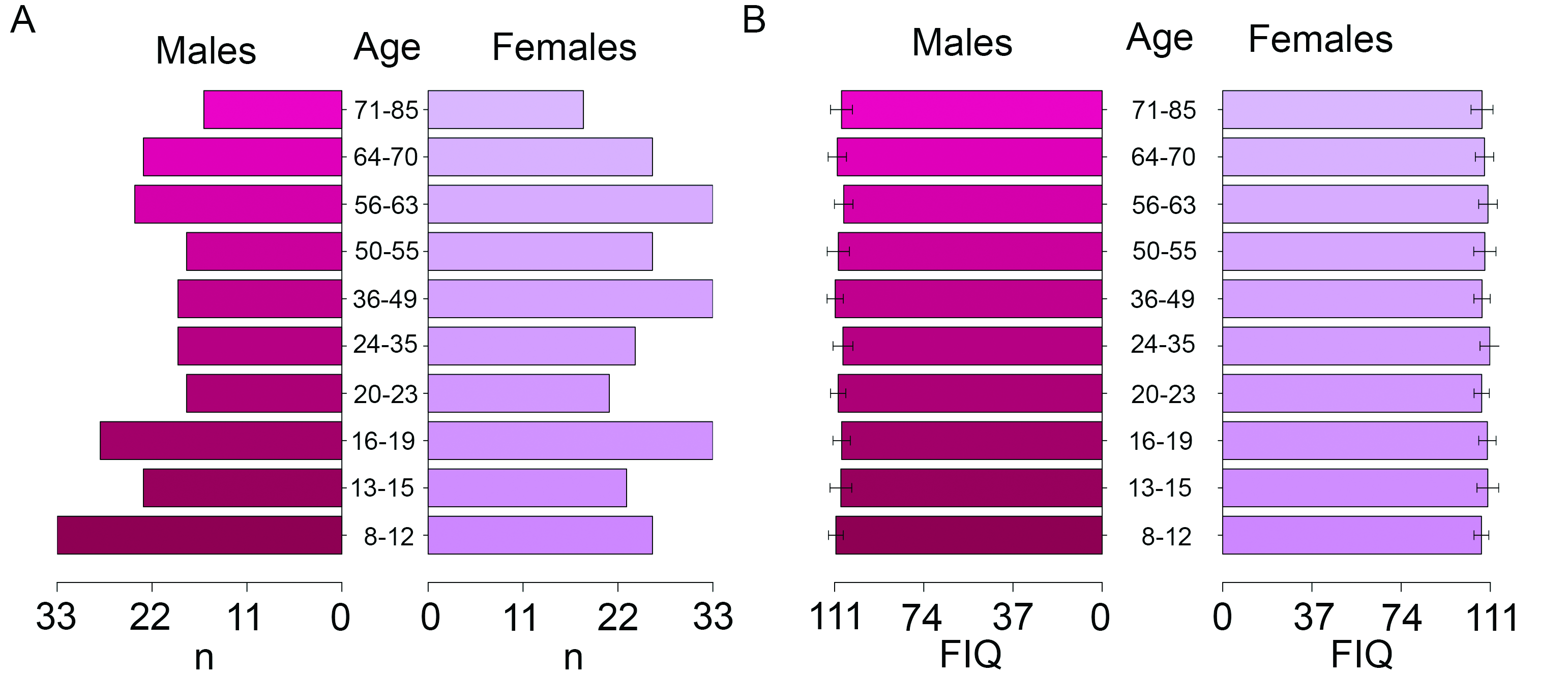


**Fig. S1**. **A**. Number of females and males for each age decile. **B.** Mean full-scale intelligent quotient (FIQ), plotted for females and males, separately, for each age decile, error bars represent standard errors.


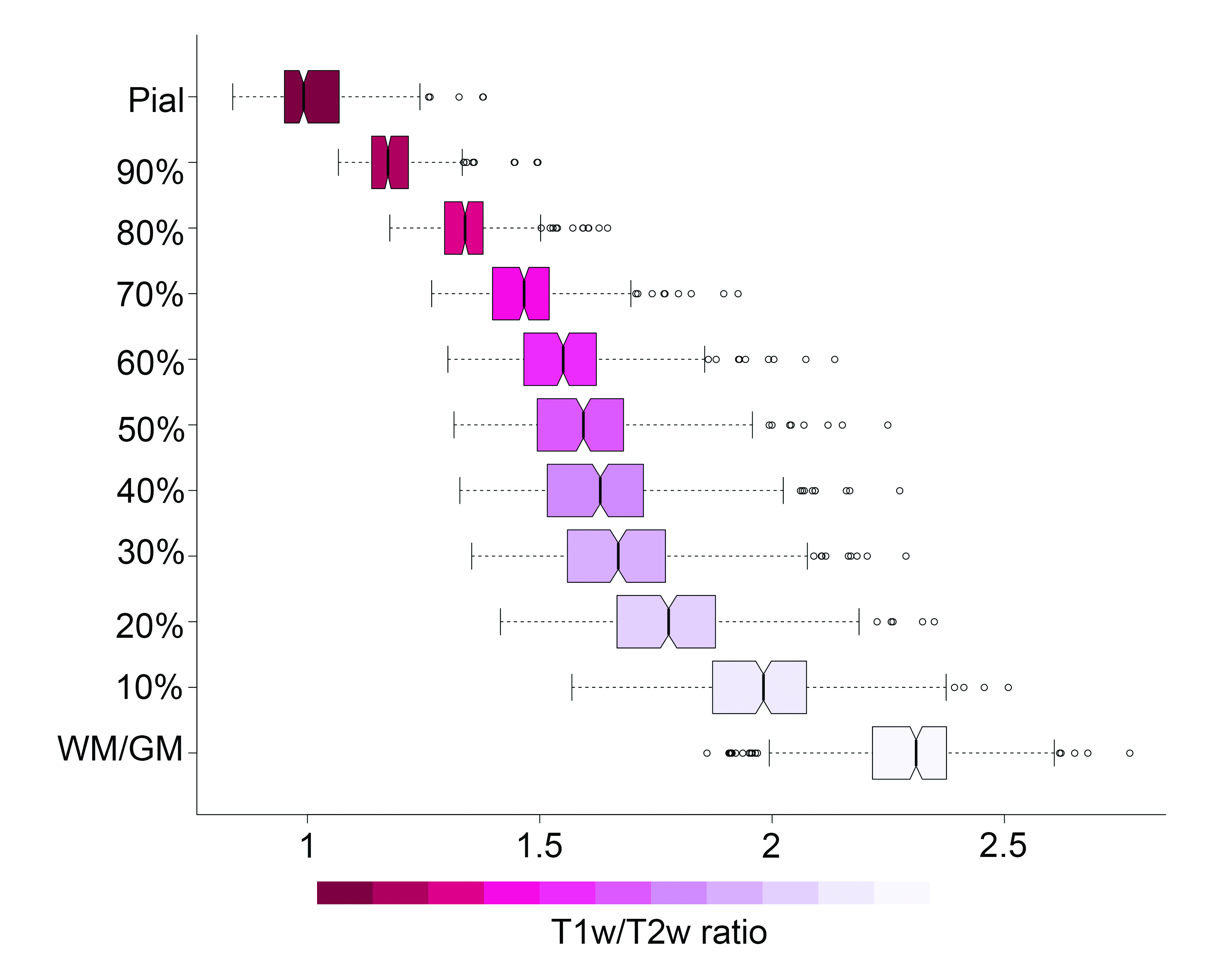


**Fig. S2**. T1w/T2w ratio values as a function of cortical depth.

**
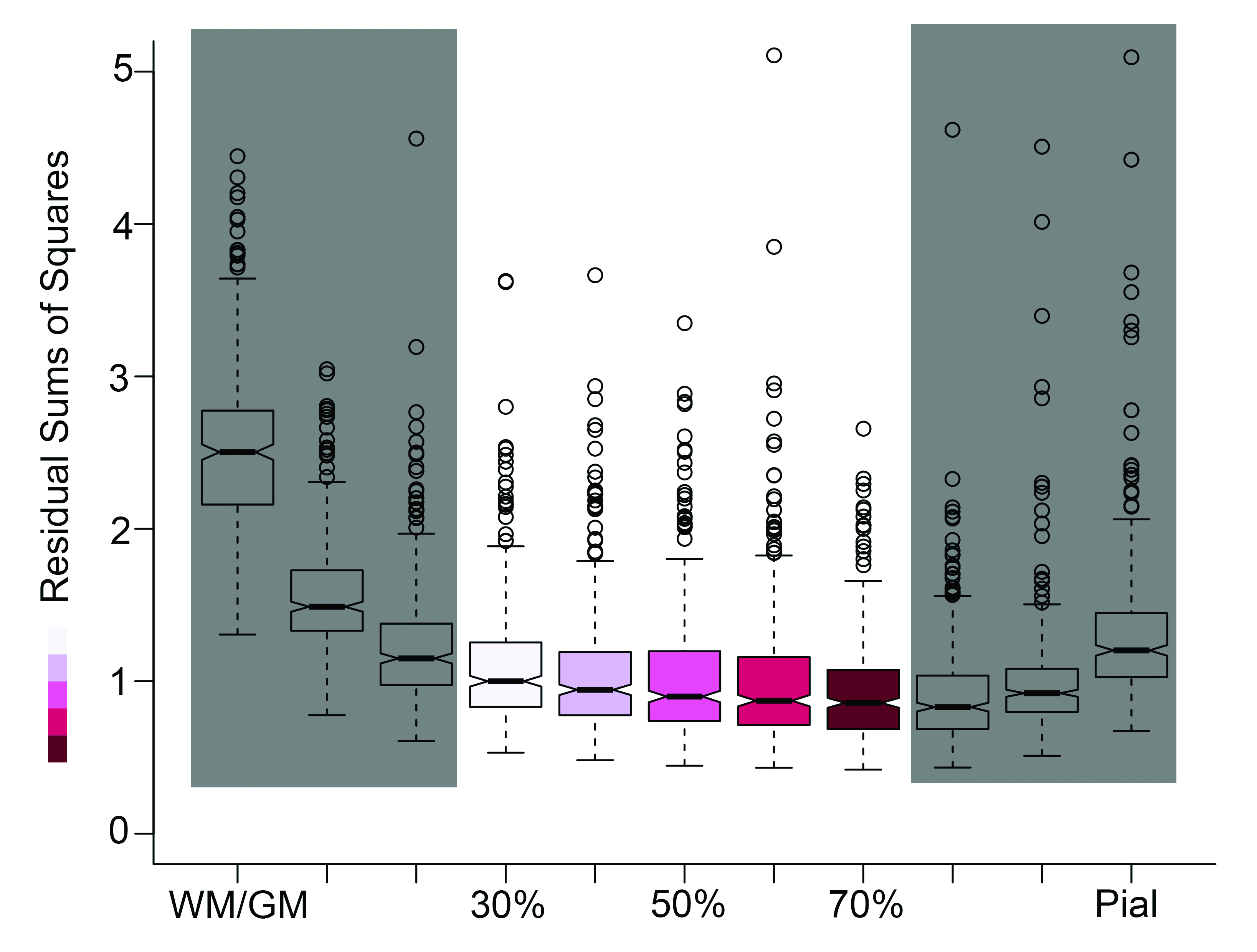
**

**Fig. S3**. Residual sum of squares from spline fitting of each of the 360 cortical regions, plotted as a function of age, at each cortical depth, across the whole sample. We only considered depths between 30% and 70% (in colour) to minimize influence of partial voluming effects from the white matter and cerebrospinal fluid, respectively (please see text for references).


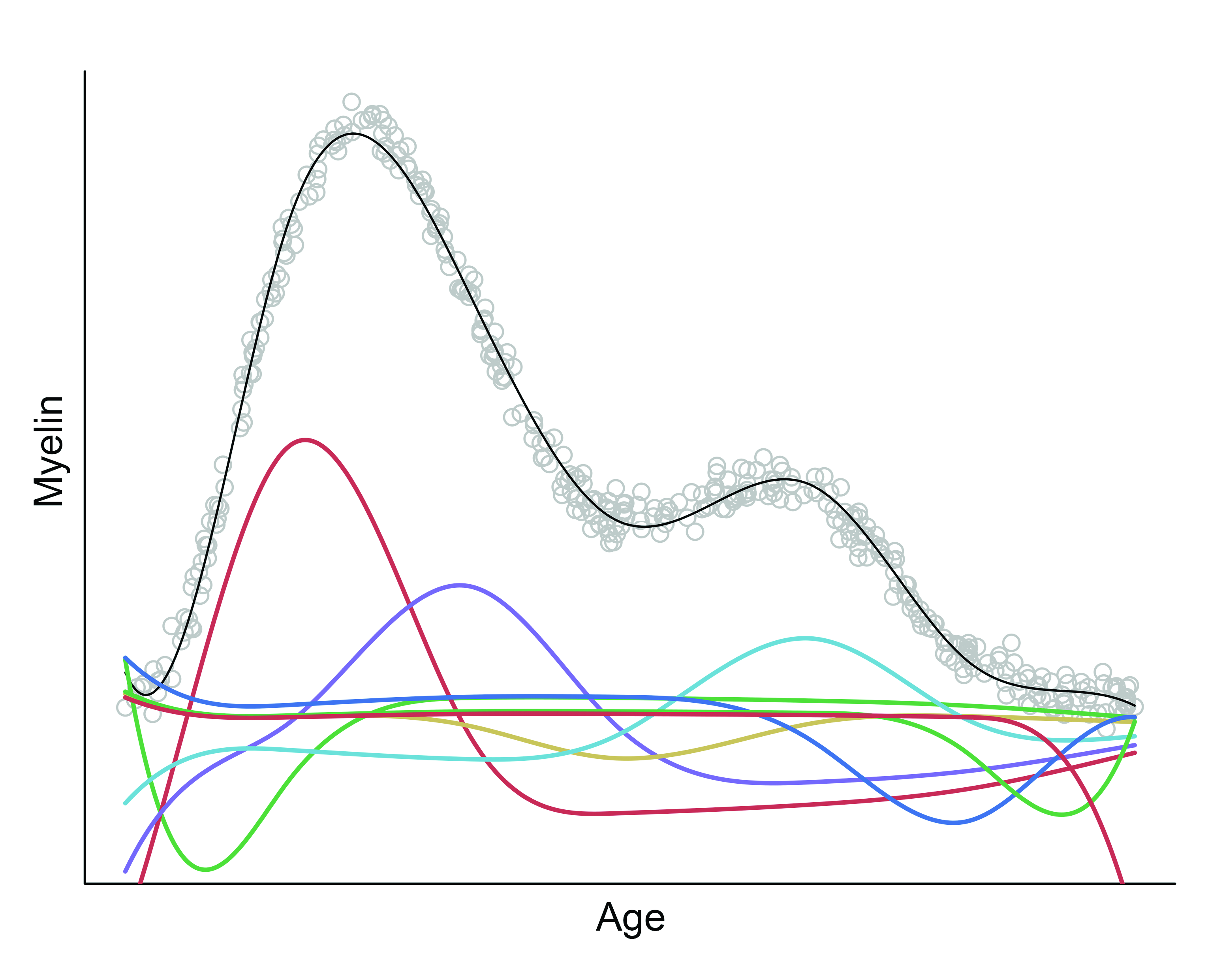


**Fig. S4**. Simulated data (grey circles) of an imaginary age-myelin relationship with a fitted cubic B-spline (black curve), which is a weighted sum of the 8 basis functions, multiplied by their respective coefficients, plotted below (coloured curves).


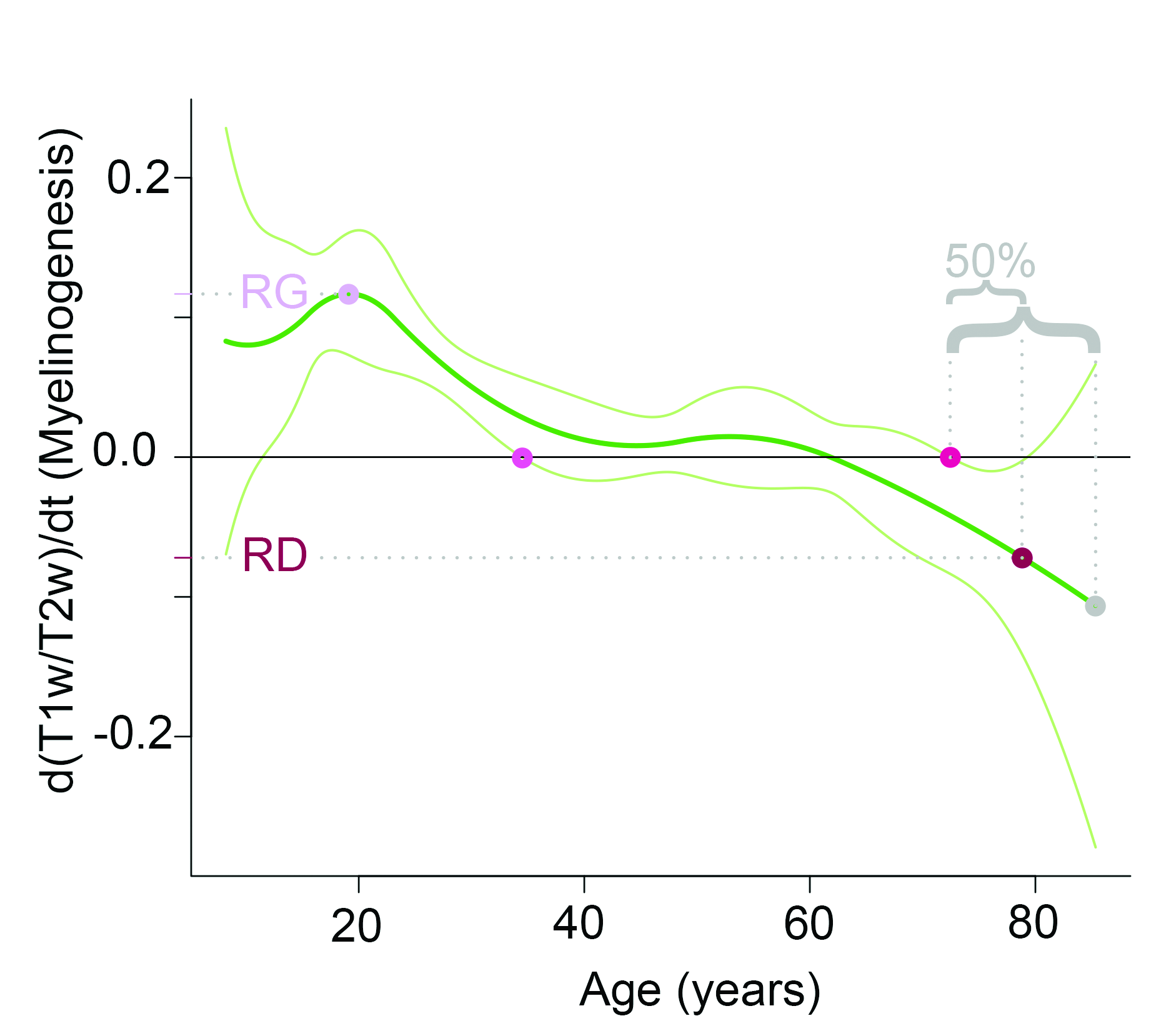


**Fig. S5**. Derivative of average curve in **Fig. 1B** (main text), showing rate of growth (RG), and rate of decline (RD) points. The rate of growth was obtained from the point of peak growth, and RD was obtained at the halfway-point (50% of the distance) between the onset of decline age and the maximum age in the sample.


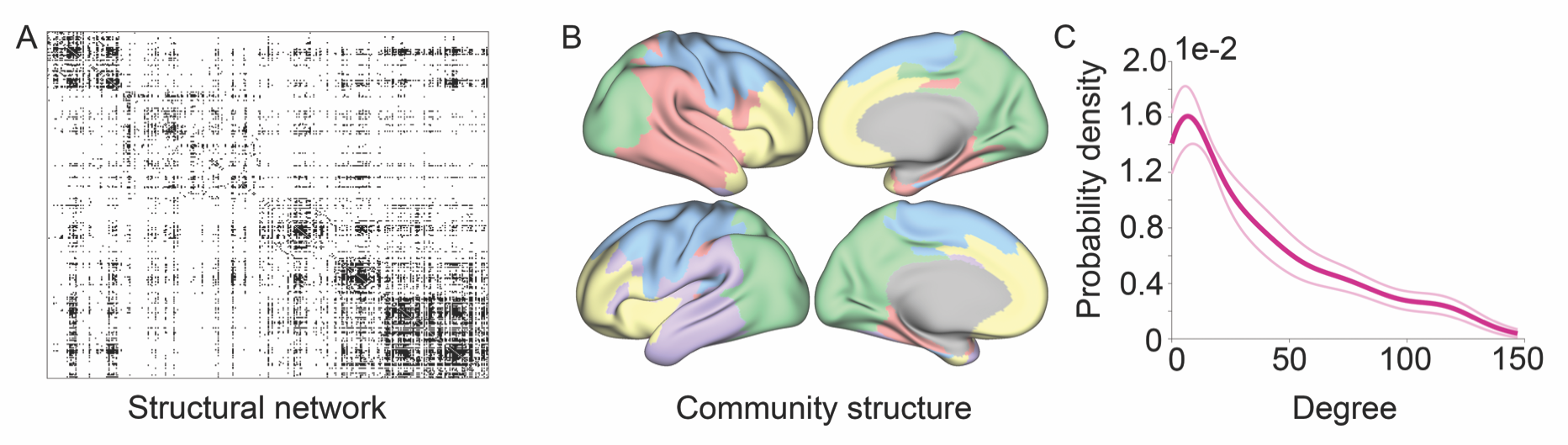


**Fig. S6**. **A**. A 360×360 structural covariance matrix based on correlations between all regions for the 103 participants aged between the point of maturity and decline, respectively, i.e. from 34 and 72 years. The matrix has been binarized, retaining 10% of the strongest connections or edges, shown in black, and sorted based on the community structure shown in **B**. **B**. Community structure, showing 5 distinct communities, or modules, represented in different colours. **C**. Probability density plot of the degree distribution. In this binary network, the degree is the number of connections (edges) per brain region (node). The 95% confidence bands (light magenta) were generated by bootstrap with replacements across participants over 1000 iterations.


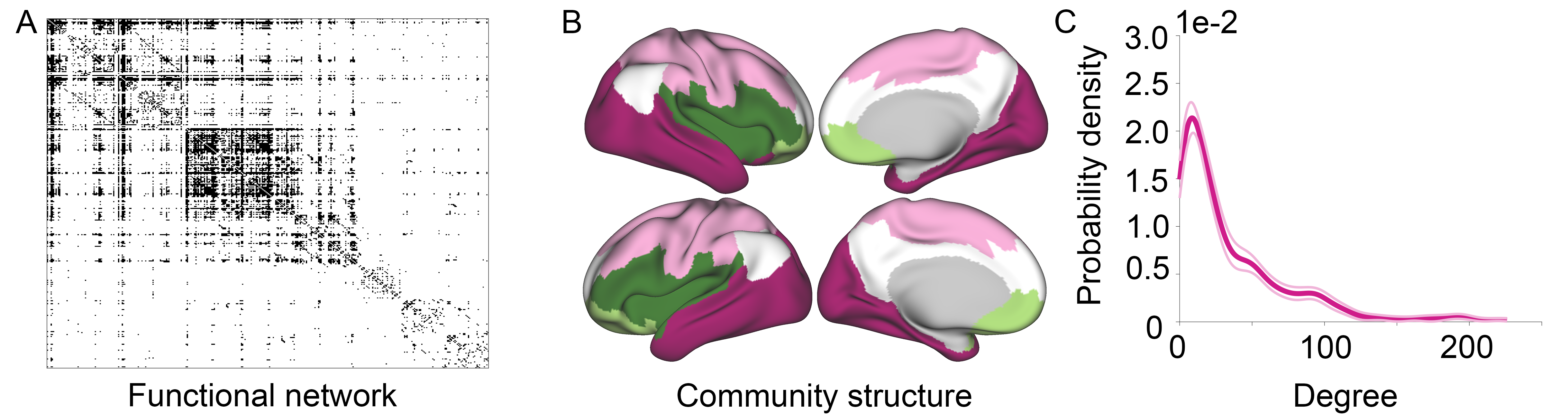


**Fig. S7**. **A**. A 360×360 functional connectivity matrix based on the average connectivity matrices of 128 participants aged from 20 and 72 years. The matrix has been binarized, retaining 10% of the strongest connections or edges, shown in black, and sorted based on the community structure shown in **B**. **B**. Community structure, showing 5 distinct communities, or modules, represented in different colours. **C**. Probability density plot of the degree distribution. In this binary network, the degree is the number of connections (edges) per brain region (node). The 95% confidence bands (light magenta) were generated by bootstrap with replacements across participants over 1000 iterations.


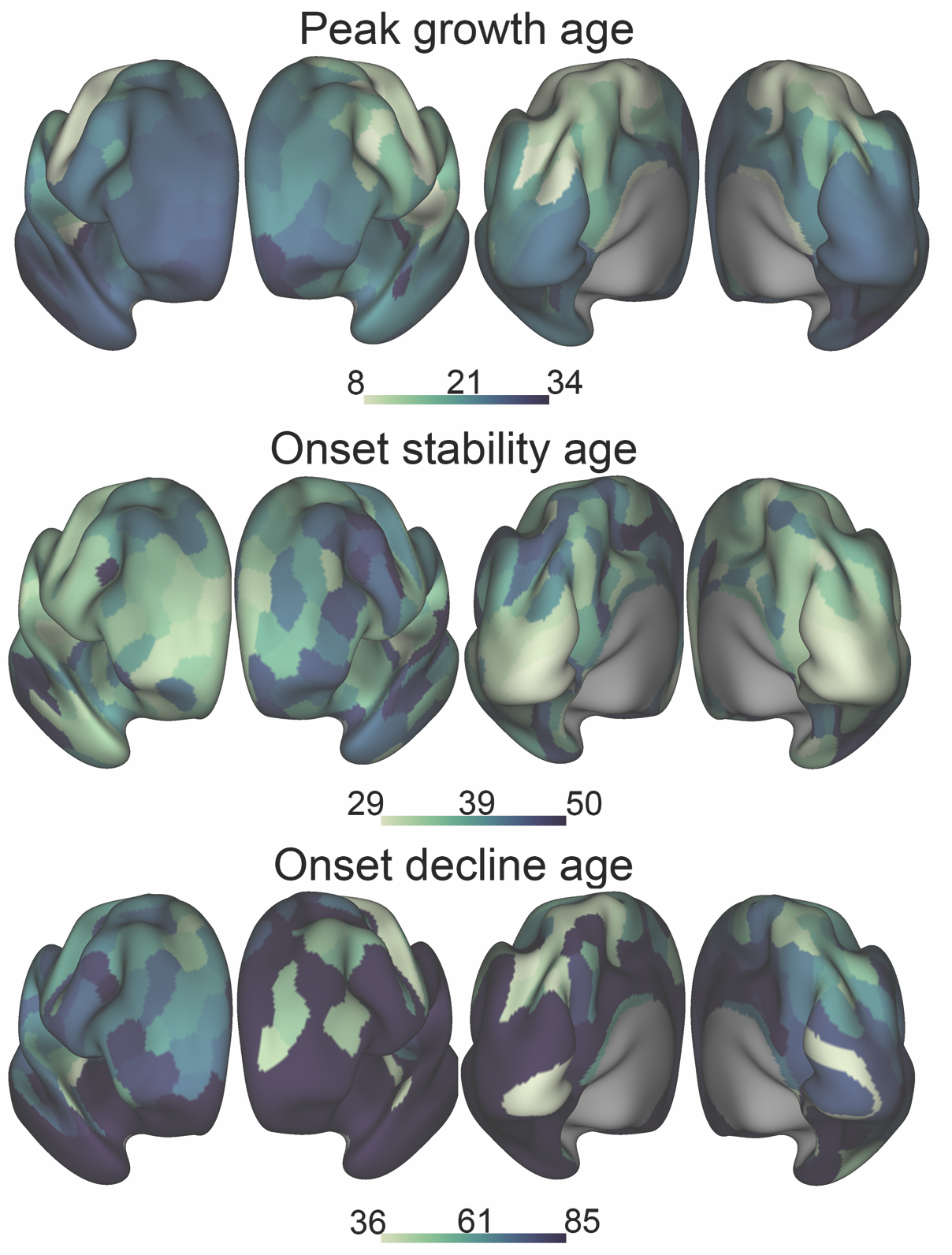


**Fig. S8**. Surface maps showing anterior and posterior views of peak growth age, onset stability age, and onset decline age (see also **Fig. 2A** in main text).


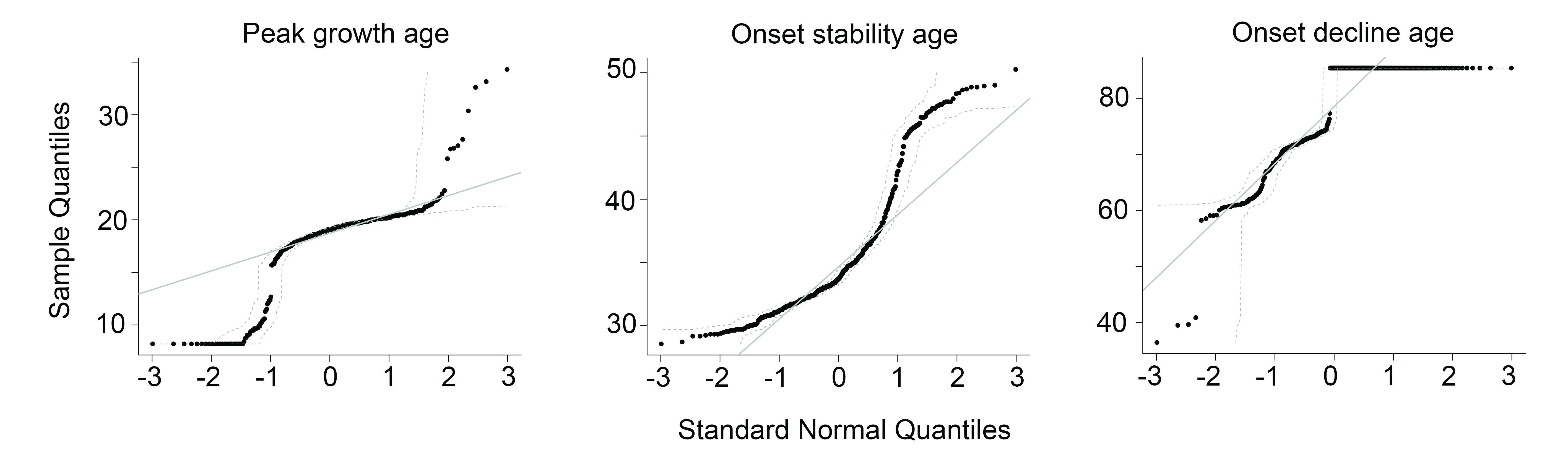


**Fig. S9**. Quantile-quantile plots for age at peak growth, age at onset, and decline onset. Solid grey line passes through the first and third quantile, and dotted grey lines represents 95% simultaneous confidence bands (obtained using the R package *extRemes*).


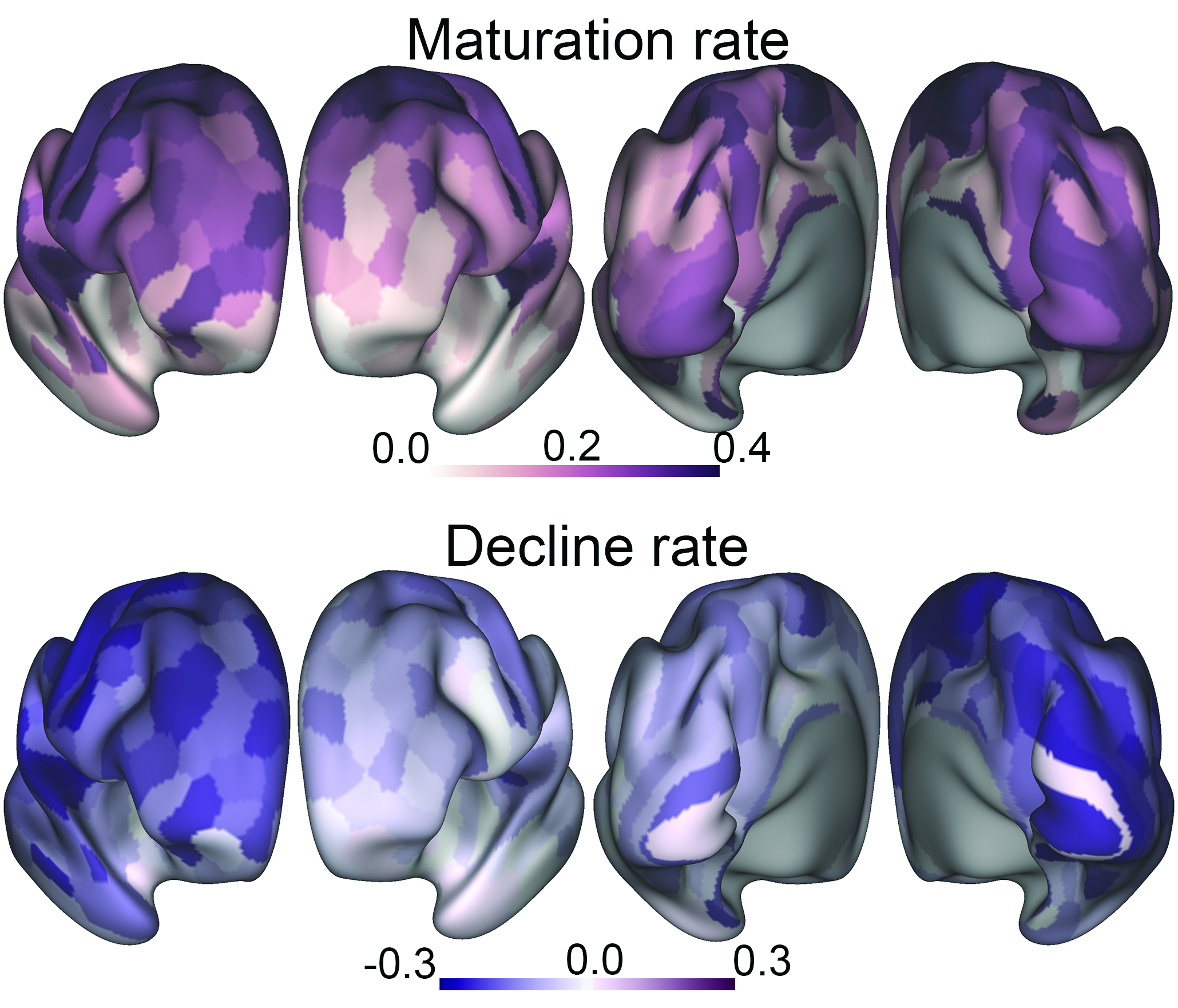


**Fig. S10**. Surface maps showing anterior and posterior views of rates of peak growth and decline (see also **Fig. 3A** in main text).


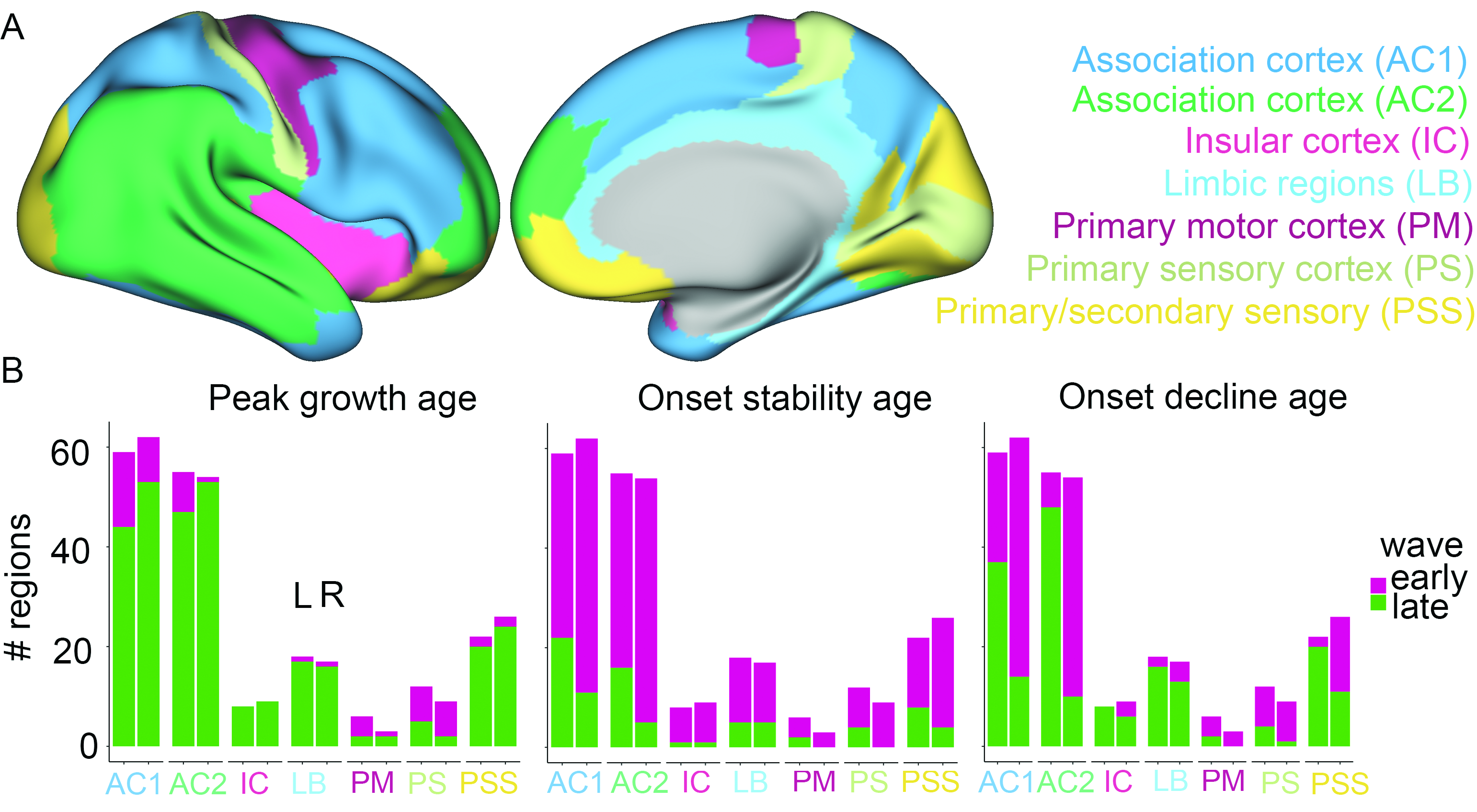


**Fig. S11. A**. Surface maps showing the 180 right hemisphere regions clustered in 7 cytoarchitectonic classes: AC1/2=association cortex 1/2, IC=insular cortex, LB=limbic cortex, PM=primary motor cortex, PS=primary sensory cortex, PSS=primary/secondary sensory cortex. **B**. Bar charts, for each class and hemisphere, showing number of regions in the early and late waves for peak growth age, onset stability age, and onset decline age. L/R = left/right hemisphere.


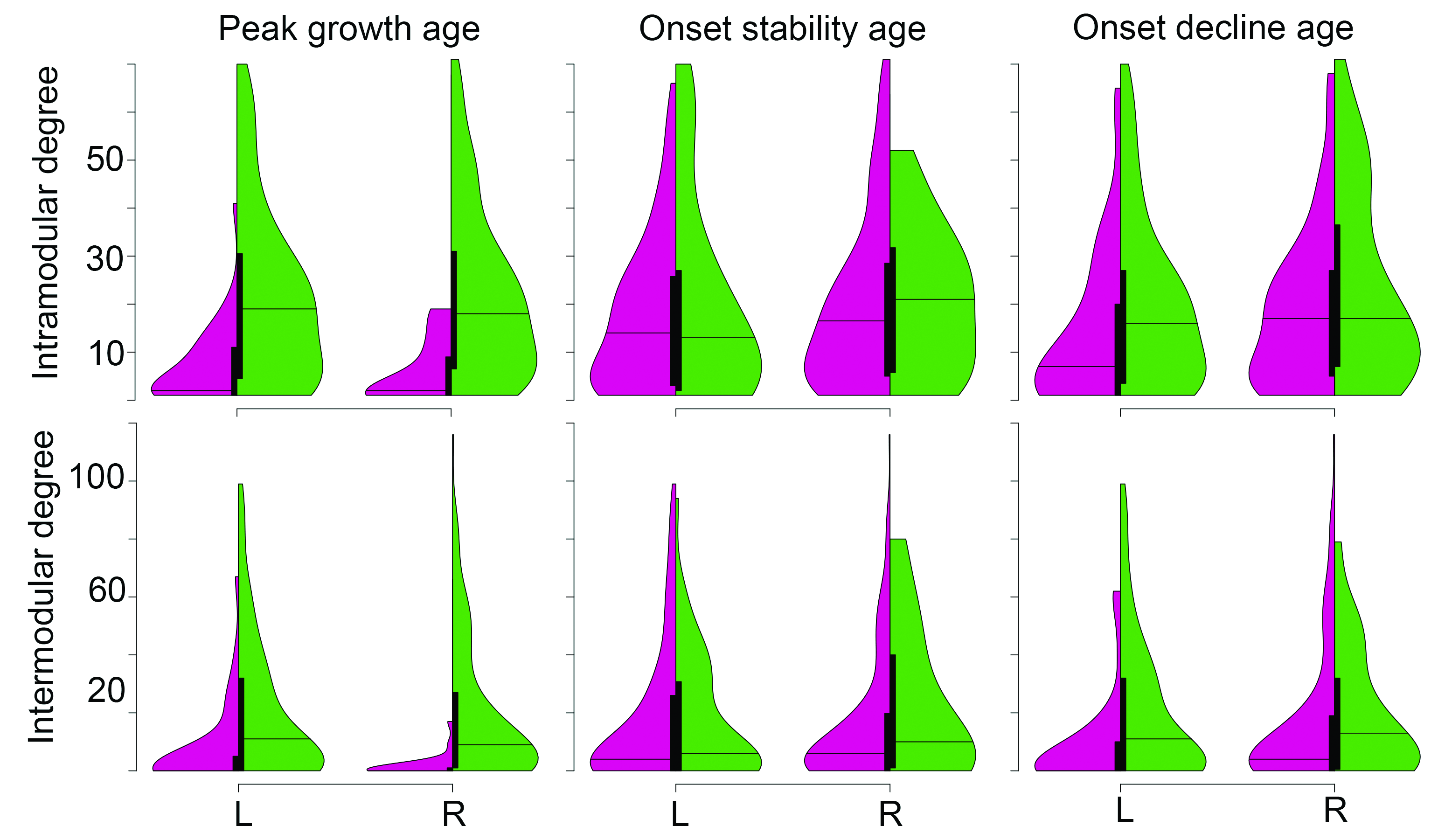


**Fig. S12**. Violin plots, split per hemisphere and early (magenta) and late (dark green) wave, for peak growth age, onset stability age, and onset decline age, showing distribution of intramodular degree (top), and intermodular degree (bottom). L, left hemisphere, R, right hemisphere. Peak growth and onset of decline occurred later in particularly global hubs (see also **Fig. 4** in main text).


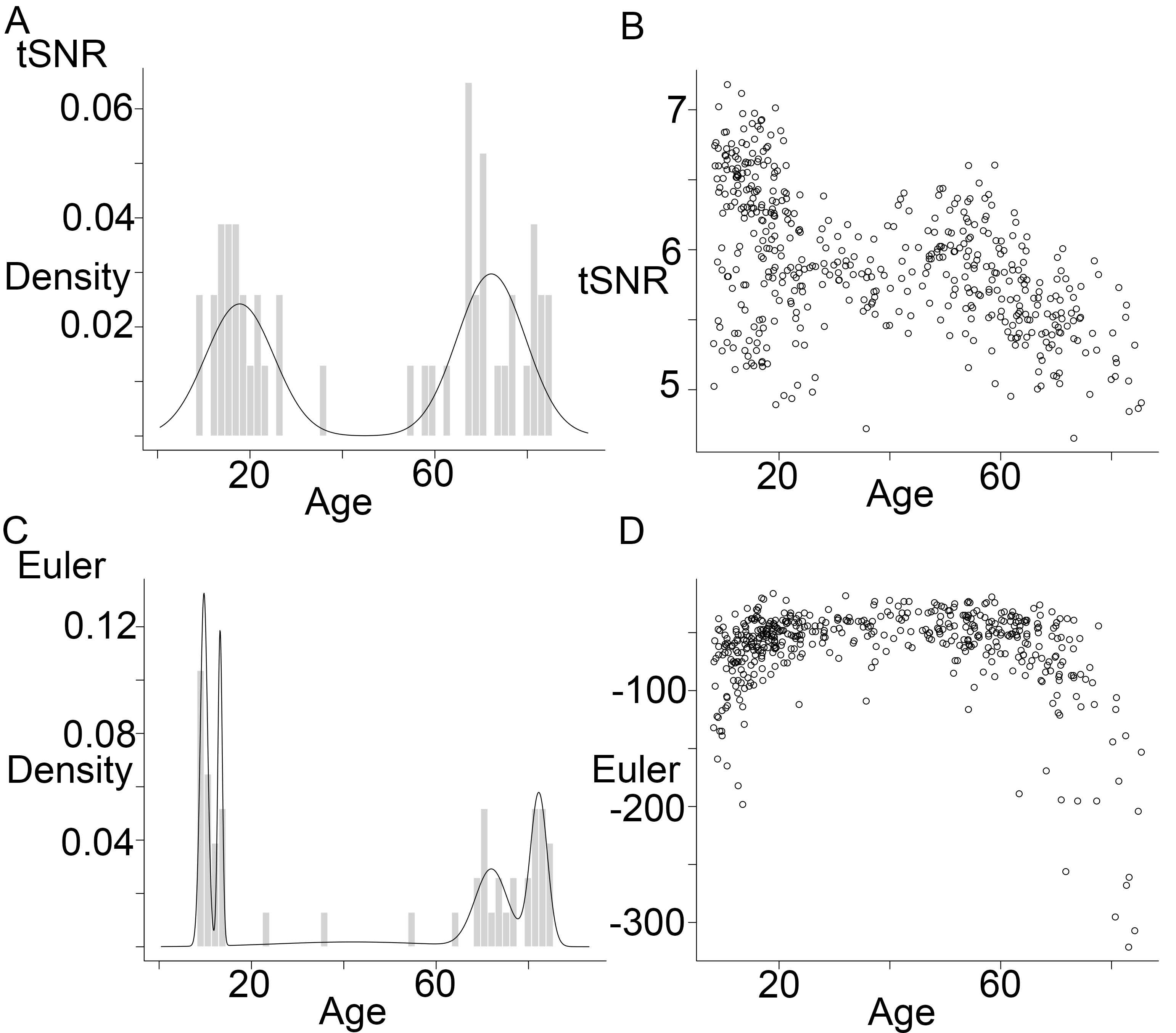


**Fig. S13**. A. Age histogram of the 49 participants with tSNR lower than the 10^th^ percentile. B. tSNR (y axis) plotted as a function of age (x axis). C. Age histogram of the 49 participants with Euler number lower than the 10^th^ percentile. B. Euler number (y axis) plotted as a function of age (x axis), one participant with Euler value of -745 (aged 79.9 years), was excluded for clarity.


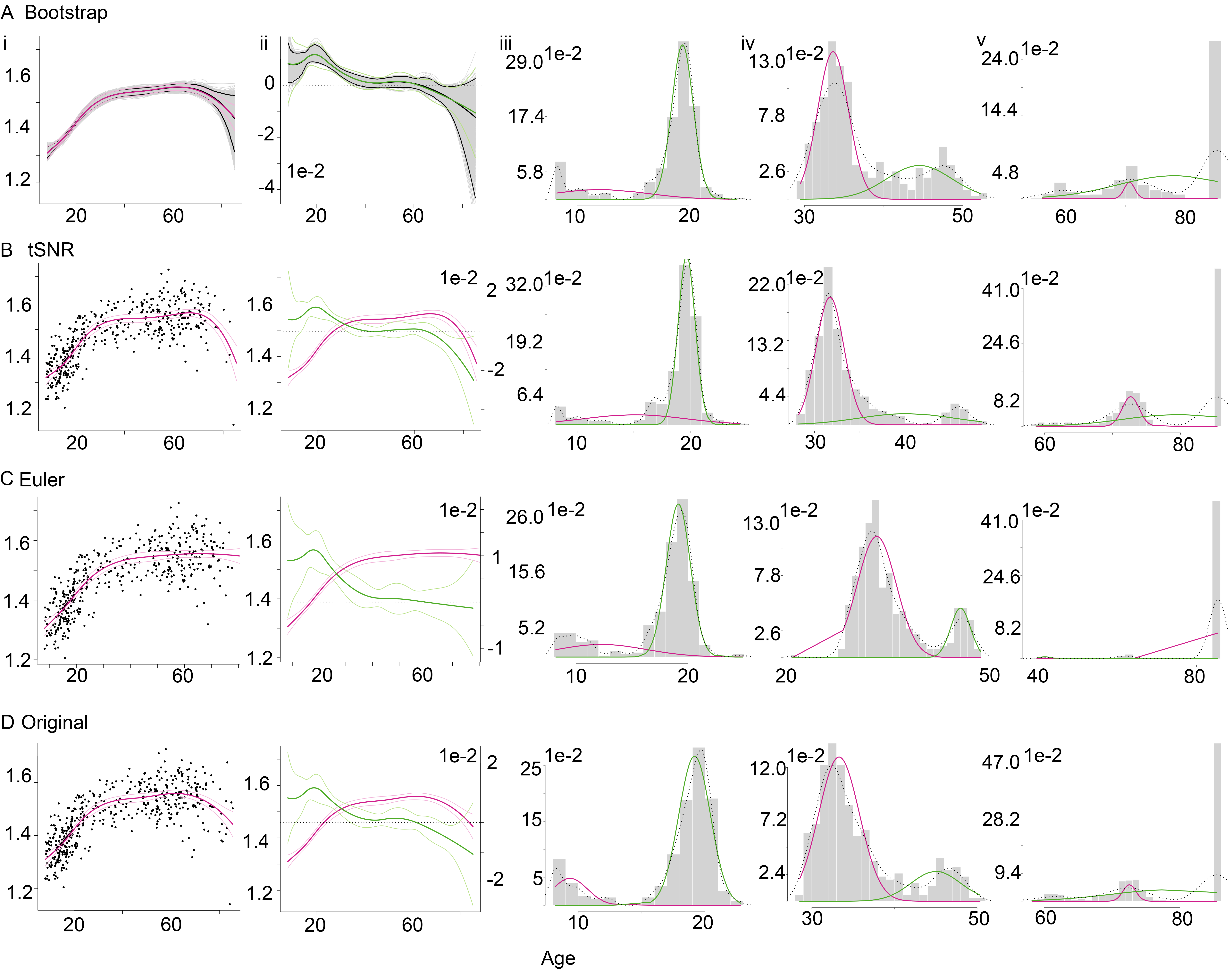


**Fig. S14**. Sensitivity analyses. Re-plotting of figures 1B, 1C, and 2B with (A) 99% confidence intervals calculated using the bootstrap method (5000 resamples with replacements), each grey line represents a spline fitted to a bootstrap sample, black lines represent the mean across bootstrap samples, and the 99% confidence intervals, respectively (for (iv), very minor artefacts (lack of smoothness) at the very beginning of the lower CI, caused artefactual early M3 in some regions, which were discarded here for simplicity), (B) a sub-sample of 435 participants excluding 10% of participants with the lowest tSNR, (C) a sub-sample of 435 participants excluding 10% of participants with the lowest Euler number. (D) The original figures plotted for ease of comparison. In column i) the y axis unit is “T1w/T2w” (as in 1B), in column ii) the y axes units are “T1w/T2w” and “d(T1w/T2w)/dt”, respectively (except for the bootstrap, which is only “d(T1w/T2w)/dt”), while in the histograms, the y axis unit is Density.

**Supplementary References**

Achard S, Delon-Martin C, Vertes PE, Renard F, Schenck M, Schneider F, Heinrich C, Kremer S, Bullmore ET. 2012. Hubs of brain functional networks are radically reorganized in comatose patients. Proc Natl Acad Sci U S A. 109:20608-20613.

Alexander-Bloch A, Giedd JN, Bullmore E. 2013. Imaging structural co-variance between human brain regions. Nat Rev Neurosci. 14:322-336.

Alexander-Bloch AF, Gogtay N, Meunier D, Birn R, Clasen L, Lalonde F, Lenroot R, Giedd J, Bullmore ET. 2010. Disrupted modularity and local connectivity of brain functional networks in childhood-onset schizophrenia. Front Syst Neurosci. 4:147.

Alexander-Bloch AF, Reiss PT, Rapoport J, McAdams H, Giedd JN, Bullmore ET, Gogtay N. 2014. Abnormal cortical growth in schizophrenia targets normative modules of synchronized development. Biol Psychiatry. 76:438-446.

Beck AT, Steer R. 1987. Beck Depression Inventory Scoring Manual. New York: The Psychological Corporation.

Benjamini Y, Yekutieli D. 2001. The control of the false discovery rate in multiple testing under dependency. Ann Stat. 29:1165-1188.

Blondel VD, Guillaume JL, Lambiotte R, Lefebvre E. 2008. Fast unfolding of communities in large networks. J Stat Mech-Theory E.

Bullmore E, Fadili J, Maxim V, Sendur L, Whitcher B, Suckling J, Brammer M, Breakspear M. 2004. Wavelets and functional magnetic resonance imaging of the human brain. Neuroimage. 23 Suppl 1:S234-249.

Dale AM, Fischl B, Sereno MI. 1999. Cortical surface-based analysis. I. Segmentation and surface reconstruction. Neuroimage. 9:179-194.

Evans AC. 2013. Networks of anatomical covariance. Neuroimage. 80:489-504.

Fischl B, Dale AM. 2000. Measuring the thickness of the human cerebral cortex from magnetic resonance images. Proc Natl Acad Sci U S A. 97:11050-11055.

Fischl B, Salat DH, Busa E, Albert M, Dieterich M, Haselgrove C, van der Kouwe A, Killiany R, Kennedy D, Klaveness S, Montillo A, Makris N, Rosen B, Dale AM. 2002. Whole brain segmentation: automated labeling of neuroanatomical structures in the human brain. Neuron. 33:341-355.

Fischl B, Salat DH, van der Kouwe AJ, Makris N, Segonne F, Quinn BT, Dale AM. 2004. Sequence-independent segmentation of magnetic resonance images. Neuroimage. 23 Suppl 1:S69-84.

Fischl B, Sereno MI, Dale AM. 1999. Cortical surface-based analysis. II: Inflation, flattening, and a surface-based coordinate system. Neuroimage. 9:195-207.

Fischl B, Sereno MI, Tootell RB, Dale AM. 1999. High-resolution intersubject averaging and a coordinate system for the cortical surface. Hum Brain Mapp. 8:272-284.

Fischl B, van der Kouwe A, Destrieux C, Halgren E, Segonne F, Salat DH, Busa E, Seidman LJ, Goldstein J, Kennedy D, Caviness V, Makris N, Rosen B, Dale AM. 2004. Automatically parcellating the human cerebral cortex. Cereb Cortex. 14:11-22.

Fjell AM, Walhovd KB, Westlye LT, Ostby Y, Tamnes CK, Jernigan TL, Gamst A, Dale AM. 2010. When does brain aging accelerate? Dangers of quadratic fits in cross-sectional studies. Neuroimage. 50:1376-1383.

Folstein MF, Folstein SE, McHugh PR. 1975. "Mini-mental state". A practical method for grading the cognitive state of patients for the clinician. J Psychiatr Res. 12:189-198.

Glasser MF, Coalson TS, Robinson EC, Hacker CD, Harwell J, Yacoub E, Ugurbil K, Andersson J, Beckmann CF, Jenkinson M, Smith SM, Van Essen DC. 2016. A multi-modal parcellation of human cerebral cortex. Nature. 536:171-178.

Glasser MF, Sotiropoulos SN, Wilson JA, Coalson TS, Fischl B, Andersson JL, Xu J, Jbabdi S, Webster M, Polimeni JR, Van Essen DC, Jenkinson M, Consortium WU-MH. 2013. The minimal preprocessing pipelines for the Human Connectome Project. Neuroimage. 80:105-124.

Glasser MF, Van Essen DC. 2011. Mapping human cortical areas in vivo based on myelin content as revealed by T1- and T2-weighted MRI. J Neurosci. 31:11597-11616.

Greve DN, Fischl B. 2009. Accurate and robust brain image alignment using boundary-based registration. Neuroimage. 48:63-72.

Grydeland H, Walhovd KB, Tamnes CK, Westlye LT, Fjell AM. 2013. Intracortical myelin links with performance variability across the human lifespan: results from T1- and T2-weighted MRI myelin mapping and diffusion tensor imaging. J Neurosci. 33:18618-18630.

Hartigan JA, Hartigan P. 1985. The dip test of unimodality. The Annals of Statistics.70-84.

Huntenburg JM, Bazin PL, Goulas A, Tardif CL, Villringer A, Margulies DS. 2017. A Systematic Relationship Between Functional Connectivity and Intracortical Myelin in the Human Cerebral Cortex. Cereb Cortex. 27:981-997.

Jenkinson M, Bannister P, Brady M, Smith S. 2002. Improved optimization for the robust and accurate linear registration and motion correction of brain images. Neuroimage. 17:825-841.

Kritzer MF, Goldman-Rakic PS. 1995. Intrinsic circuit organization of the major layers and sublayers of the dorsolateral prefrontal cortex in the rhesus monkey. J Comp Neurol. 359:131-143.

Lerch JP, Worsley K, Shaw WP, Greenstein DK, Lenroot RK, Giedd J, Evans AC. 2006. Mapping anatomical correlations across cerebral cortex (MACACC) using cortical thickness from MRI. Neuroimage. 31:993-1003.

Levitt JB, Lewis DA, Yoshioka T, Lund JS. 1993. Topography of pyramidal neuron intrinsic connections in macaque monkey prefrontal cortex (areas 9 and 46). J Comp Neurol. 338:360-376.

Marra G, Wood SN. 2012. Coverage properties of confidence intervals for generalized additive model components. Scandinavian Journal of Statistics. 39:53-74.

Patel AX, Kundu P, Rubinov M, Jones PS, Vertes PE, Ersche KD, Suckling J, Bullmore ET. 2014. A wavelet method for modeling and despiking motion artifacts from resting-state fMRI time series. Neuroimage. 95:287-304.

Polimeni JR, Fischl B, Greve DN, Wald LL. 2010. Laminar analysis of 7T BOLD using an imposed spatial activation pattern in human V1. Neuroimage. 52:1334-1346.

Roalf DR, Quarmley M, Elliott MA, Satterthwaite TD, Vandekar SN, Ruparel K, Gennatas ED, Calkins ME, Moore TM, Hopson R, Prabhakaran K, Jackson CT, Verma R, Hakonarson H, Gur RC, Gur RE. 2016. The impact of quality assurance assessment on diffusion tensor imaging outcomes in a large-scale population-based cohort. Neuroimage. 125:903-919.

Robinson EC, Jbabdi S, Glasser MF, Andersson J, Burgess GC, Harms MP, Smith SM, Van Essen DC, Jenkinson M. 2014. MSM: a new flexible framework for Multimodal Surface Matching. Neuroimage. 100:414-426.

Rosen AFG, Roalf DR, Ruparel K, Blake J, Seelaus K, Villa LP, Ciric R, Cook PA, Davatzikos C, Elliott MA, Garcia de La Garza A, Gennatas ED, Quarmley M, Schmitt JE, Shinohara RT, Tisdall MD, Craddock RC, Gur RE, Gur RC, Satterthwaite TD. 2018. Quantitative assessment of structural image quality. Neuroimage. 169:407-418.

Rubinov M, Sporns O. 2010. Complex network measures of brain connectivity: uses and interpretations. Neuroimage. 52:1059-1069.

Scholtens LH, de Reus MA, de Lange SC, Schmidt R, van den Heuvel MP. 2016. An MRI Von Economo - Koskinas atlas. Neuroimage.

Segonne F, Dale AM, Busa E, Glessner M, Salat D, Hahn HK, Fischl B. 2004. A hybrid approach to the skull stripping problem in MRI. Neuroimage. 22:1060-1075.

Shinn M, Romero-Garcia R, Seidlitz J, Vasa F, Vertes PE, Bullmore E. 2017. Versatility of nodal affiliation to communities. Sci Rep. 7:4273.

Smith SM, Jenkinson M, Woolrich MW, Beckmann CF, Behrens TE, Johansen-Berg H, Bannister PR, De Luca M, Drobnjak I, Flitney DE, Niazy RK, Saunders J, Vickers J, Zhang Y, De Stefano N, Brady JM, Matthews PM. 2004. Advances in functional and structural MR image analysis and implementation as FSL. Neuroimage. 23 Suppl 1:S208-219.

Solari SV, Stoner R. 2011. Cognitive consilience: primate non-primary neuroanatomical circuits underlying cognition. Front Neuroanat. 5:65.

Sporns O, Betzel RF. 2016. Modular Brain Networks. Annu Rev Psychol. 67:613-640.

Tamnes CK, Ostby Y, Fjell AM, Westlye LT, Due-Tonnessen P, Walhovd KB. 2010. Brain maturation in adolescence and young adulthood: regional age-related changes in cortical thickness and white matter volume and microstructure. Cereb Cortex. 20:534-548.

Tamnes CK, Walhovd KB, Dale AM, Ostby Y, Grydeland H, Richardson G, Westlye LT, Roddey JC, Hagler DJ, Jr., Due-Tonnessen P, Holland D, Fjell AM. 2013. Brain development and aging: overlapping and unique patterns of change. Neuroimage. 68:63-74.

Tantrum J, Murua A, Stuetzle W editors. Assessment and pruning of hierarchical model based clustering, Proceedings of the ninth ACM SIGKDD international conference on Knowledge discovery and data mining; 2003:ACM. 197-205 p.

Triarhou LC. 2007. The Economo-Koskinas atlas revisited: cytoarchitectonics and functional context. Stereotact Funct Neurosurg. 85:195-203.

van den Heuvel MP, Scholtens LH, Feldman Barrett L, Hilgetag CC, de Reus MA. 2015. Bridging Cytoarchitectonics and Connectomics in Human Cerebral Cortex. J Neurosci. 35:13943-13948.

van der Kouwe AJW, Benner T, Salat DH, Fischl B. 2008. Brain morphometry with multiecho MPRAGE. Neuroimage. 40:559-569.

Vertes PE, Rittman T, Whitaker KJ, Romero-Garcia R, Vasa F, Kitzbichler MG, Wagstyl K, Fonagy P, Dolan RJ, Jones PB, Goodyer IM, Consortium N, Bullmore ET. 2016. Gene transcription profiles associated with inter-modular hubs and connection distance in human functional magnetic resonance imaging networks. Philos Trans R Soc Lond B Biol Sci. 371.

Wechsler D. 1999. Wechsler Abbreviated Scale of Intelligence. San Antonio, TX: The Psychological Corporation.

Westlye LT, Walhovd KB, Bjornerud A, Due-Tonnessen P, Fjell AM. 2009. Error-related negativity is mediated by fractional anisotropy in the posterior cingulate gyrus--a study combining diffusion tensor imaging and electrophysiology in healthy adults. Cereb Cortex. 19:293-304.

Wood SN. 2006. Generalized Additive Models: An Introduction with R. Boca Raton, FL: CRC Press.

Wood SN. 2011. Fast stable restricted maximum likelihood and marginal likelihood estimation of semiparametric generalized linear models. J R Stat Soc B. 73:3-36.

Woolrich MW, Jbabdi S, Patenaude B, Chappell M, Makni S, Behrens T, Beckmann C, Jenkinson M, Smith SM. 2009. Bayesian analysis of neuroimaging data in FSL. Neuroimage. 45:S173-186.

Xu L, Bedrick EJ, Hanson T, Restrepo C. 2014. A comparison of statistical tools for identifying modality in body mass distributions. Journal of Data Science. 12:175-196.

Yarkoni T, Poldrack RA, Nichols TE, Van Essen DC, Wager TD. 2011. Large-scale automated synthesis of human functional neuroimaging data. Nat Methods. 8:665-670.
